# Forensic biomarkers of lethal traumatic brain injury

**DOI:** 10.1007/s00414-022-02785-2

**Published:** 2022-02-28

**Authors:** Johann Zwirner, Rachel Kulakofsky, Antonia Fitzek, Ann Sophie Schröder, Simone Bohnert, Heike Franke, Thomas Renné, Rexson Tse, Benjamin Ondruschka

**Affiliations:** 1 Department of Anatomy, University of Otago, Dunedin, New Zealand; 2 Institute of Legal Medicine, University Medical Center Hamburg-Eppendorf, Hamburg, Germany; 3 Institute of Legal Medicine, University of Leipzig, Leipzig, Germany; 4 Institute of Forensic Medicine, University of Wuerzburg, Wuerzburg, Germany; 5 Rudolf Boehm Institute of Pharmacology and Toxicology, University of Leipzig, Leipzig, Germany; 6 Institute of Clinical Chemistry and Laboratory Medicine, University Medical Center Hamburg-Eppendorf (UKE), Hamburg, Germany; 7 Irish Centre for Vascular Biology, School of Pharmacy and Biomolecular Sciences, Royal College of Surgeons in Ireland, Dublin, Ireland; 8 Center for Thrombosis and Hemostasis (CTH), Johannes Gutenberg University Medical Center, Mainz, Germany; 9 Department of Forensic Pathology, LabPLUS, Auckland City Hospital, Auckland, New Zealand

**Keywords:** Biomarker, Cause of death, Forensic biochemistry, Survival time, Time since death, Traumatic brain injury, Post-mortem

## Abstract

Traumatic brain injury (TBI) is a major cause of death and its accurate diagnosis is an important concern of daily forensic practice. However, it can be challenging to diagnose TBI in cases where macroscopic signs of the traumatic head impact are lacking and little is known about the circumstances of death. In recent years, several post-mortem studies investigated the possible use of biomarkers for providing objective evidence for TBIs as the cause of death or to estimate the survival time and time since death of the deceased. This work systematically reviewed the available scientific literature on TBI-related biomarkers to be used for forensic purposes. Post-mortem TBI-related biomarkers are an emerging and promising resource to provide objective evidence for cause of death determinations as well as survival time and potentially even time since death estimations. This literature review of forensically used TBI-biomarkers revealed that current markers have low specificity for TBIs and only provide limited information with regards to survival time estimations and time since death estimations. Overall, TBI fatality-related biomarkers are largely unexplored in compartments that are easily accessible during autopsies such as urine and vitreous humor. Future research on forensic biomarkers requires a strict distinction of TBI fatalities from control groups, sufficient sample sizes, combinations of currently established biomarkers, and novel approaches such as metabolomics and mi-RNAs.

## Introduction

As defined by the US Centers for Disease Control and Prevention, a traumatic brain injury (TBI) describes a disruption of the brain’s normal function caused by bumps, blows, jolts, or penetrating head injuries [1]. TBI considerably contributes to the global injury burden and in light of a growing population, the absolute number of TBIs is expected to grow further [2]. A lethal outcome occurs in approximately a quarter to a third of patients who suffer a severe TBI, which is about the same percentage compared to the ones that fully recover from a severe traumatic head impact [3]. A TBI-related death most often results from intentional self-harm (33%), followed by unintentional falls (28%) and motor vehicle accidents (19%) [4]. Hence, it is not surprising that TBI is an important topic for forensic pathologists with cases ranging from suicidal head banging [5] to homicidal head blows [6]. Especially, when macroscopic signs of head impacts such as contusions, bleedings, or lacerations are lacking, it can be challenging to determine a TBI as the sole or contributing cause of death [7]. Post-mortem biochemical analyses could be a promising objective resource for forensic pathologists to diagnose lethal TBIs as the cause of death. Forensic biochemical investigations are already described and widely used for the cause of death determination of hypothermia, ketoacidosis, myocardial infarction, drowning, or anaphylaxis [8]. From ante-mortem studies, it is known that several biomarkers show significant differences following traumatic head impacts compared to atraumatic controls [9, 10]. On that basis, several forensic groups have explored the potential to use those TBI biomarkers for forensic purposes in a post-mortem setting [7, 11–13]. However, forensic expectations on TBI biomarkers as well as the conditions under which the samples are obtained considerably differ from ante-mortem clinical practice. Clinically used TBI biomarkers provide information on the diagnosis, prognosis, and treatment efficiency of TBIs [14]. In contrast, forensic pathologists expect additional objective data on TBI survival time estimations or time since death estimations [15, 16]. Ante-mortem, TBI biomarkers are determined in blood or cerebrospinal fluid (CSF), which are sampled from living individuals under aseptic conditions [17]. Contrary to that, the body fluids for the determination of TBI biomarkers in forensic cases are sampled from dead and often at least partly putrefied individuals during forensic autopsies. Post-mortem changes and sampling conditions raise the question of whether forensic biochemical investigations can provide any valuable information at all [7, 18]. Contrary to the clinical setting, practically all tissues of the human body can be used to determine TBI biomarkers in forensic investigations. However, as post-mortem reference values for different causes of death are lacking, their potential value for forensic investigations related to TBI fatalities has to be explored from scratch. This given work provides an up-to-date review of the post-mortem biochemistry of lethal TBIs including information on their value for forensically relevant topics such as cause of death determinations, survival time estimations, and time since death estimations. Also, it will be compiled whether these TBI biomarkers are relevantly influenced by factors such as age, sex, hemolysis, perimortem rescue procedures, or storage conditions. Thus far, a forensically focused TBI biomarker review is not available. The following eight biomarkers were chosen to be presented in this review: S100 calcium-binding protein B (S100B), neuron-specific enolase (NSE), glial fibrillary acidic protein (GFAP), interleukin-6 (IL-6), brain-derived neurotrophic factor (BDNF), and microtubule-associated protein tau (MAPT), which were selected as these are well-known candidate fluid biomarkers related to TBI pathophysiology [19]. Furthermore, lactate dehydrogenase (LDH), ferritin, and neutrophil gelatinase-associated lipocalin (NGAL) were chosen to be presented here based on previous own post-mortem studies of the authors. However, the search strategy was not limited to the selected biomarkers to avoid the risk of missing important others.

## Materials/Methods

The here performed review of post-mortem TBI biomarkers contains the following two components: (i) a systematic component of previous post-mortem studies to detect a fatal TBI including the following biomarkers: S100B, NSE, GFAP, IL-6, LDH, Ferritin, BDNF, NGAL, and MPAT; (ii) data from peer-reviewed studies that summarize information from clinical studies on the respective biomarker or laboratory analyses, which were not part of the strategic search but provide important context for the forensic investigations. This literature review compiles the following information for each of the abovementioned biomarkers:

- Molecular weight **-** what is the molecular weight of the respective marker?
- Expression - where is the biomarker expressed within the human body?
- Function - which function does the marker serve within the human body (as far as this has been answered to date)?
- Cause of death determination - does the biomarker allow to significantly differentiate lethal TBIs and non-TBI control cases?
- Survival time estimation - does the biomarker discriminate different survival times between the traumatic head impact and the death on a statistically significant level?
- Post-mortem interval correlation - was the marker discriminative with regards to the post-mortem interval (PMI) on a statistically significant level? The PMI refers to the time between the death of the cadaver and the autopsy, in which the tissues undergo alterations such as degradation or putrefaction.
- Reason for biomarker level change within compartment - which mechanism underlies the significantly different biomarker concentration between TBI fatalities and controls in the respective compartment?
- Age- or sex-dependence - did the biomarker correlate with the age at death or the sex of the deceased in the respective compartment on a statistically significant level?
- Hemolysis index (H-index) dependence - did the biomarker level correlate with the hemolysis index of the fluid sample on a statistically significant level?
- Influence of rescue procedures or intensive care procedures - was the biomarker level in the respective compartment significantly different, if the deceased was subjected to rescue (e.g., cardiopulmonary resuscitation attempt) or intensive care unit procedures (e.g., neurosurgical intervention)?
- Comparison to clinical biomarker levels - how were the TBI-related biomarker levels measured in a forensic setting compared to known clinical values of the identical compartment?
- In vitro freeze–thaw-cycle influence - did the in vitro biomarker concentration within a forensically relevant compartment correlate with the number of applied freeze–thaw cycles on a statistically significant level?
- In vitro biomarker stability - does the in vitro measured biomarker concentration within a forensically relevant compartment change over time on a statistically significant level? *P*-values of 0.05 or less were considered to be statistically significant.

**Fig. 1:**
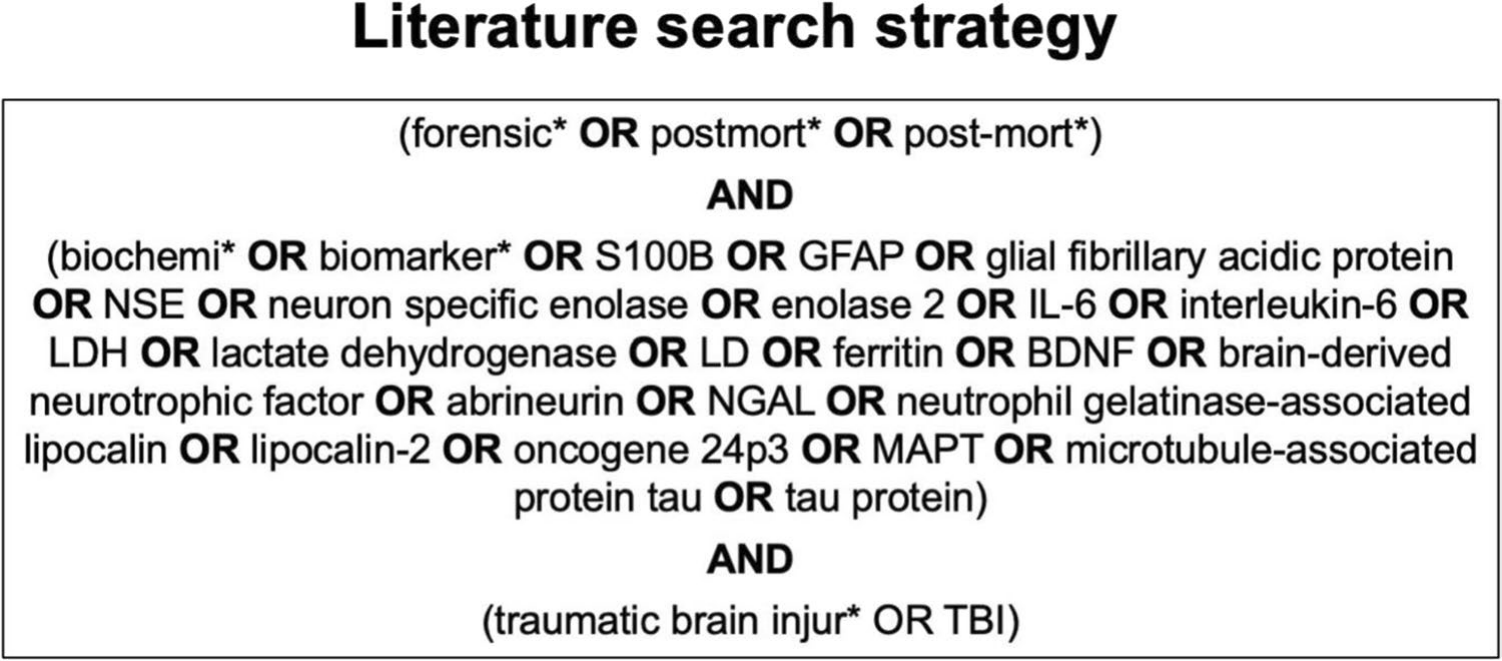
The search strategy for the systematic part of this literature review is depicted

The information for the points cause of death determination, survival time estimation, age- or sex-dependence, H-index, and the post-mortem biomarker levels for the comparison to the clinical values were extracted from PubMed-listed forensic studies. These were searched up until August 2021 according to the Preferred Reporting Items for Systematic Reviews and Meta-analyses (PRISMA) guidelines [20] (Fig. 1). Initially, the articles were screened by their title and abstract. If the title and/or abstract revealed that the selected TBI-related biomarkers were measured in post-mortem tissues, the full text was sought for retrieval and assessed for eligibility. Then, the reference lists of the respective papers were screened. The following inclusion criteria were defined: (i) TBI as the cause of death, (ii) study must contain a control group, (iii) only studies on humans, and (iv) only peer-reviewed original works. The systematic part of the literature review was independently performed by two authors (JZ and RK) according to the inclusion criteria that are listed in Fig. 1. A third author (BO) checked the results for accuracy and decided, whether information that the two authors (JZ and RK) could not agree on should be included.

## Results

**Fig. 2:**
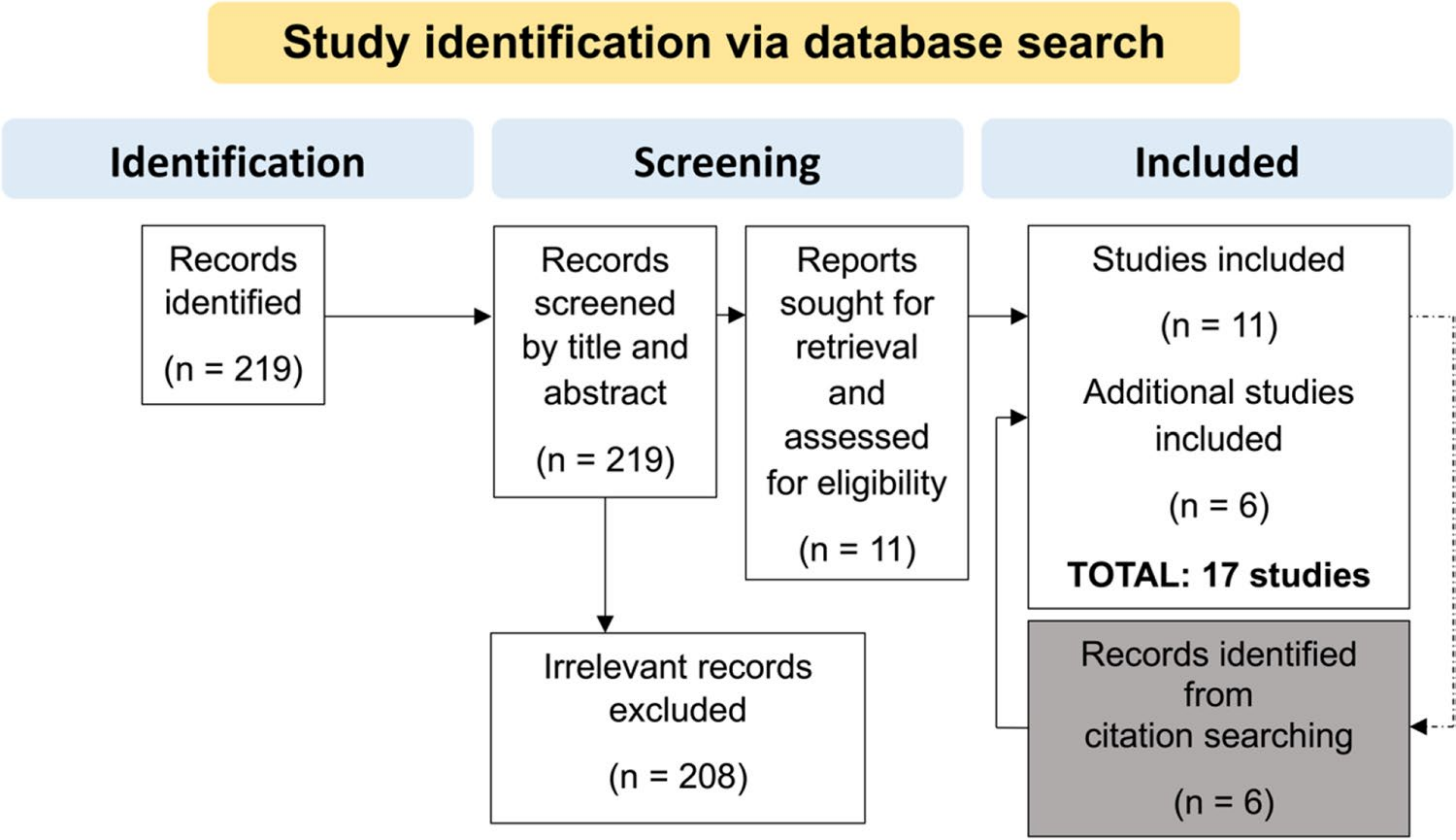
PRISMA flow chart for the methodology undertaken for the screening of relevant literature based on Moher et al. [20]

**Fig. 3:**
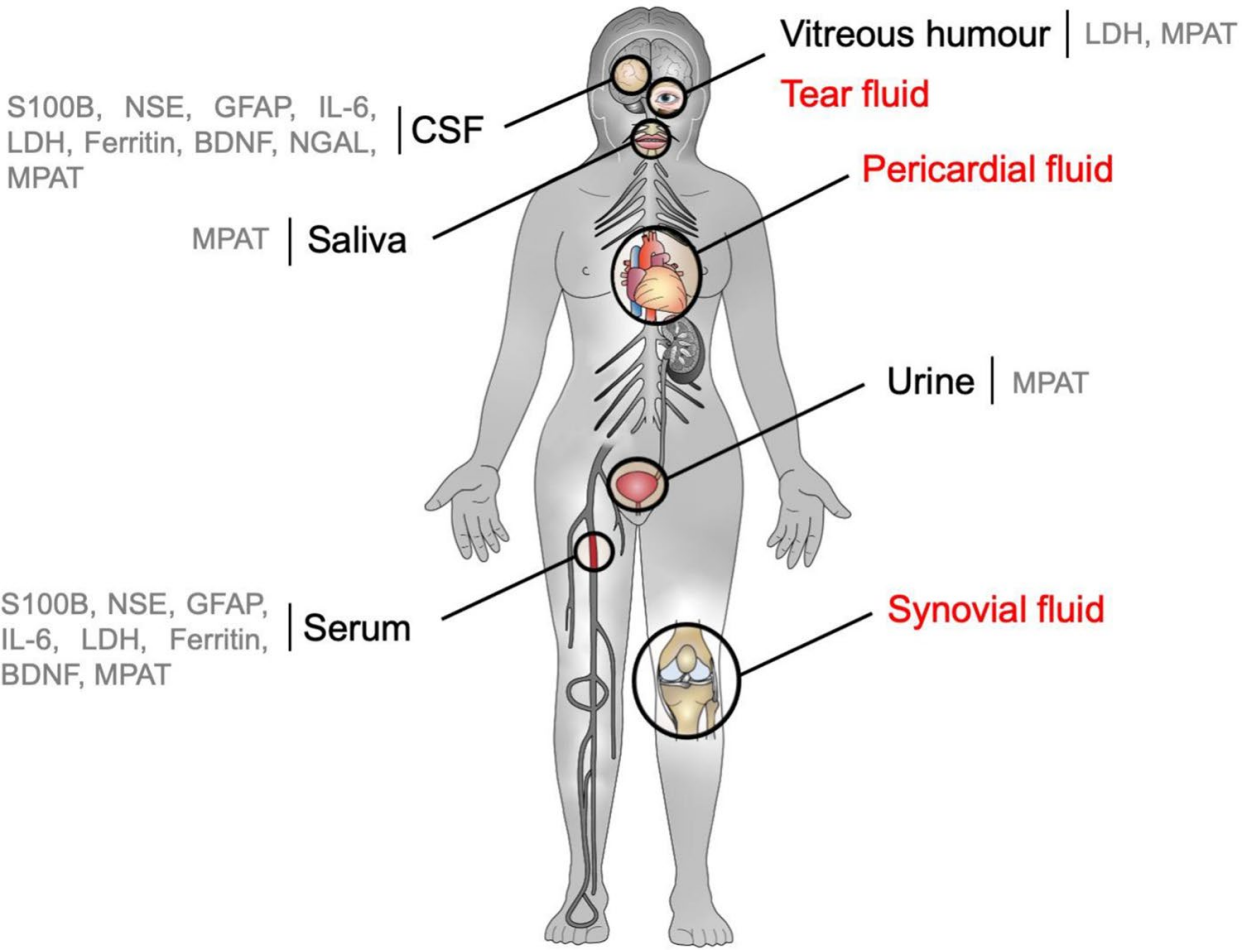
The sampling fluids to measure TBI-related biomarker concentrations in forensic studies are depicted. Available fluids that can be sampled during autopsy but have not been used for TBI-related biomarker measurements so far are depicted in red color

**Fig. 4:**
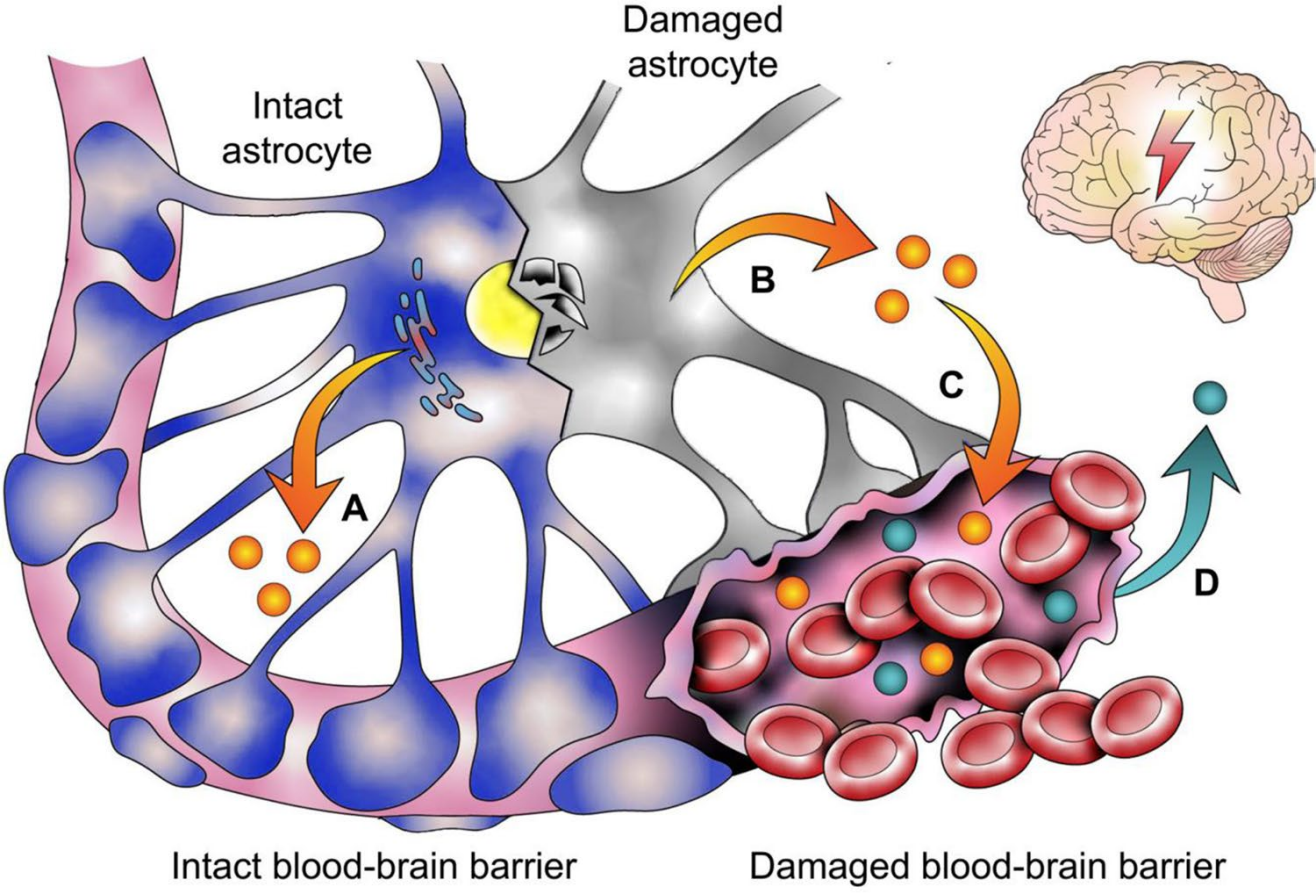
Several methods for the change in biomarker concentrations following traumatic head impacts are depicted for CSF and blood (exemplified on astrocytes). In response to a traumatic head impact, biomarkers can be secreted from intact astrocytes (**A**) or released from damaged astrocytes (**B**). Blood–brain barrier disruptions then cause an increase of biomarkers in the blood (**C**). Also, elevated biomarkers from the periphery could leak into the CSF via the disrupted blood–brain barrier (**D**)

A total of 17 studies were identified from the literature search (Fig. 2). Of these, six studies reported results dealing with IL-6 [21–26]; five with GFAP [11, 13, 24, 26, 27], LDH [23, 24, 26, 28, 29], and S100B [7, 26, 30–32]; four with NSE [24, 26, 30, 31]; three with BDNF [11, 24, 26], MAPT [13, 33, 34], and ferritin [23, 24, 26]; and two with NGAL [11, 26]. Information regarding cause of death determination and survival time estimation are presented below. The rest of the extracted data is compiled in Tables 1, 2, and 3. The used body fluids for the measurement of TBI-related biomarkers in previous studies and the hypotheses that explain the biomarker alterations after the traumatic head impact are depicted in Figs. 3 and 4.

**Table 1:** A summary of the selected forensically used traumatic brain injury (TBI) biomarkers is given. R values are only provided if the *p*-value of that correlation was significant (≤ 0.05) and the values were stated in the related studies

		S100		NSE		GFAP		IL-6	
Molecular weight [kDa]		10–12 [35]		78 [36]		50 [37]		21–26 [38]	
Expression		Astrocytes [39], (potentially) neurons after TBI [32, 40], Schwann cells [41], pituitary cells [42], stellate cells, follicular cells, folliculostellate cells [43]		1.6% of the total soluble human brain protein [44], smooth muscle cells (aorta, uterus, prostate), erythrocytes, lymphocytes, platelets [45]		Astrocytes [46], neurons (in TBI [47], Alzheimer’s disease [48] and tuberous sclerosis [49]), Schwann cells [50], bone marrow [51], spleen [51]		Monocytes [52], neurons [16], astrocytes [16, 53], microglia [54], skeletal muscle [55], fibroblasts, mesenchymal cells, endothelial cells [56]	
Functions		Cell proliferation, differentiation, migration, apoptosis inhibition, astrocyte activation after injury [57], neuronal survival enhancement [58], neurogenesis [59], cell line proliferation [60], apoptosis [61]		Axonal transport and homoeostasis maintenance after injury [62], neuronal survival, neuronal differentiation, neurite regeneration [63, 64]		Maintenance of astrocyte stability, reactive astrogliosis, glial scar formation, blood brain barrier integrity [65]		Immune regulation, hematopoiesis, inflammation, oncogenesis [66], bone metabolism, neural development [67], astrocyte differentiation [68]	
Reason for biomarker level change within compartment after traumatic head impact		Hypothesis for CSF: diffusion via cellular breakdown or breakdown of BBB [31, 69] Blood: transport from CSF via glymphatic system [35]		Hypothesis for blood: transport from CSF via glymphatic system [35]		Hypothesis: upregulation of GFAP following head impact depending on brain swelling [70], brain parenchyma destruction causes leakage into CSF [71]		Hypothesis: increased mRNA expression in post-mortem brain samples suggests active upregulation after TBI [72, 73]; serum: diffusion via trauma-induced BBB disruption [74]	
Age-dependency		CSF: no [30, 31] Serum: no [7, 30, 31]		CSF: no [30, 31] Serum: no [30, 31]		CSF and serum: no [11]		CSF and serum: no [11]	
Sex-dependency		CSF: no [30, 31] Serum: no [7, 30, 31]		CSF: no [30, 31] Serum: no [30, 31]		CSF and serum: no [11]		CSF and serum: no [11]	
H-index-dependency		CSF: *r* = 0.31 [30], *r* = 0.24 [31] Serum: *r* = 0.28 [30], *r* = 0.29 [31]		CSF: *r* = 0.20 [30], *r* = 0.36 [31] Serum: *r* = 0.38 [30], *r* = 0.08 [31]		CSF: strong positive [11] Serum: no [11, 27]		CSF: *r* = 0.75 [23] Serum: no [23]	
Intensive care procedure/rescue procedure-dependency		CSF and serum: no [30]		CSF and serum: no [30]		CSF and serum: no [11]		CSF: *r* ≥ 0.31 [23] Serum: 0.21 ≤ *r* ≤ 0.44 [23]	
In vitro freeze–thaw-cycle-dependency		Serum: stable for at least one cycle at − 80 °C [75]		CSF and serum: stable for at least one cycle at − 80 °C [76]		CSF: levels decreased by 50% after two cycles Serum: stable for 5 cycles at − 80 °C [77]		CSF: stable for at least two -70 °C cycles [78] Serum: stable for at least four − 20 °C cycles [79]	
In-vitro biomarker stability		Serum: at least 24 h at room temperature [75], at least 3 days at 4–5 °C [80], at least 9 months at − 80 °C [81]		CSF: 6 months at − 80 °C [76] Serum: 9 months at − 80 °C [76]		CSF: stable for 7 days at − 70 °C and 4 °C but decreased at room temperature [71, 80]		CSF: 8 years at − 70 °C [82] Serum: 21 days at 4, 20, and 30 °C, 11 days at 40 °C [79]	
Post-mortem interval correlation		CSF: *r* = 0.37 [30], *r* = 0.43 [31], no [7] Serum: *r* = 0.50 [30], *r* = 0.46 [31]		CSF: *r* = 0.25 [30], *r* = 0.18 [31] Serum: *r* = 0.56 [30], *r* = 0.32 [31]		CSF: no [11] Serum: no [11, 24]		CSF: no [23] Serum: no [23, 24]	
TBI CSF ante-mortem	TBI CSF post-mortem	ca. 11.80 ng/ml* [9]	5,470 ± 1,242 ng/ml [30]	n/a	9,235 ± 2,300 ng/ml [30]	5.5 ± 6.1 ng/ml^a^ [83]	ca. 700 ng/ml* [11]	1,100–2,200 pg/ml* [10]	3541 pg/ml [23]
Control CSF ante-mortem	Control CSF post-mortem	0.08 ± 0.003 ng/ml [9]	1,895 –4,392 ng/ml [30]	17.3 ± 4.6 ng/ml [84]	1,059–1,787 ng/ml [30]	< 0.01 ng/ml^a^ [83]	ca. 90–800 ng/ml [11]	ca. 0 pg/ml* [10]	43–122 pg/ml [23]
TBI serum ante-mortem	TBI serum post-mortem	0.026 ng/ml* [9]	583 ± 98 ng/ml [30]	7–13 ng/ml [85]	672 ± 113 ng/ml [30]	1.924 ng/ml^a^ [86]	501 ng/ml [11]	218.79 ± 56.45 pg/ml^b^ [87]	800 pg/ml [23]
Control serum ante-mortem	Control serum post-mortem	0.0003 ± 0.0001 ng/ml [9]	1,895–4,392 ng/ml [30]	8.7 ± 3.9 ng/ml [84]	388–672 ng/ml [30]	0.058 ng/ml^a^ [86] (undetectable in 123/135 cases)	77–733 ng/ml [11]	1.72 ± 0.26 pg/ml^b^ [87]	240–804 pg/ml [23]

^*^ Value read from graph; *a*, pediatric study cohort (age range 2–17 years); *b*, plasma value; *c*, sampling time not specified; *d*, TBI survival time not stated; *n/a*, not available; *r*, correlation index

**Table 2:** A summary of the selected forensically used traumatic brain injury (TBI) biomarkers (LDH. Ferritin, BDNF, NGAL) is given. R values are only provided if the *p*-value of that correlation was significant (≤ 0.05) and the values were stated in the related studies

		LDH		Ferritin		BDNF		NGAL	
**Molecular weight [kDa]**		140 [88]		500 [89]		26–28 [90, 91]		25 [92]	
Expression		Almost all tissues including neurons and astrocytes with high concentrations in the liver, muscle, and kidney [93]		Ubiquitous in the human body, predominantly cytosolic, small percentages in serum and secretary fluids [94], neurons, glial cells [95]		Astrocytes [96], neurons, microglia, oligodendroglia [91], platelets, plasma [97], visceral epithelia [98]		Neutrophils, bone marrow, trachea, lung, stomach, salivary gland, appendix, colon, prostate, and uterus [99, 100], neurons [101], (reactive) astrocytes [100]	
Functions		Glucose restoration during gluconeogenesis, single-stranded DNA metabolism [102]		Ensures solution of iron atoms as a “nanobox protein” [94]		Neuronal differentiation, development, maintenance, survival and regeneration [103], synaptic plasticity and memory functions [104, 105]		Stabilizer for the iron/siderophore complex (iron scavenger), CNS cell differentiation, invasion, migration, survival, or death [106]	
Reason for biomarker level change within compartment after traumatic head impact		Hypothesis for CSF: Leakage from CNS cells [28, 107], serum: diffusion via trauma-induced BBB disruption [108]		Upregulation of ferritin-H-chain in brain following head impact [95] Hypothesis for CSF: ferritin elevations due to secretion by macrophages/microglia [109], transudation from blood via disrupted BBB [109, 110] or released from cytosol of damaged CNS cells [109]		Hypothesis: upregulation following head impact as increased mRNA levels were observed in rodent model [111]		Upregulation in brain following TBI [101] Hypotheses for CSF: NGAL diffuses via disrupted BBB, cerebral production post injury is secondary to circulatory NGAL [101]	
Age-dependency		CSF and serum: no [23]		CSF and serum: no [23]		CSF and serum: no [11]		CSF: no [11]	
Sex-dependency		CSF and serum: no [23]		CSF and serum: no [23]		CSF and serum: no [11]		CSF: no [11]	
H-index-dependency		CSF: *r* = 0.72 [23] Serum: *r* = 0.33 [23]		CSF: *r* = 0.76 [23] Serum: no [23]		CSF: *r* = 0.54 [11] Serum: no [11]		CSF: *r* = 0.59 [11]	
Intensive care procedure/rescue procedure-dependency		CSF: *r* ≥ 0.31 [23] Serum: 0.21 ≤ *r* ≤ 0.44 [23]		CSF: *r* ≥ 0.31 [23] Serum: 0.21 ≤ *r* ≤ 0.44 [23]		CSF: *r* = 0.29 [11] Serum: no [11]		CSF: *r* = 0.34 [11]	
In vitro freeze–thaw-cycle-dependency		Serum: stable for 5 cycles at − 20 °C [112], stable for 10 cycles at − 80 °C [113], unstable after first cycle at − 196 °C [114]		Serum: stable for 10 cycles at − 80 °C [113]		Serum and plasma: at least 2 cycles at − 80 °C [115]		Serum: stable for at least 3 cycles at − 80 °C [116] Plasma: stable for at least 10 cycles at − 80 °C [117]	
In vitro biomarker stability		Serum: unstable between − 20 °C and 4 °C, but stable at − 30 °C and 25 °C for at least 14 days [118], unstable if sample separation was delayed by 1 h [114]		Serum: stable for 5 days when stored at 4 °C [119] and 5 years at − 80 °C [120] Plasma: stable for 5 days when stored at 4 °C [119]		Serum and plasma: stable for at least 2 h at room temperature and 6 months at − 80 °C [115]		Serum: decreased when stored at 25 °C, but remained stable at 4 °C between 9 h and 7 days after sampling [116] Plasma: stable for 11 months at − 80 °C [117]	
Post-mortem interval correlation		CSF: *r* = 0.43 [23], yes [28] Serum: *r* = 0.41 [23] Vitreous humor: no [28]		CSF: *r* = 0.48 [23] Serum: *r* = 0.39 [23]		CSF: no [11] Serum: no [24]		CSF: no [11]	
TBI CSF ante-mortem	TBI CSF post-mortem	5.45–15.87 µkat/l [121]	60.9 µkat/l [23]	n/a	4,700 ng/ml [23]	ca. 250 pg/ml* [122]	ca. 450 pg/ml* [11]	n/a	1,500 ng/ml* [11]
Control CSF ante-mortem	Control CSF post-mortem	0.35 ± 0.15 µkat/l [121]	4.7–19.3 µkat/l [23]	3.9 ± 1.8 ng/ml [123]	1,320–1,870 ng/ml [23]	140 ± 20 pg/ml [122]	ca. 5–10 pg/ml* [11]	n/a	80 ng/ml* [11]
TBI serum ante-mortem	TBI serum post-mortem	5–24.83 µkat/l [108, 121]	47.4 µkat/l [23]	324.8 ± 21.1 ng/ml [124]	2,830 ng/ml [23]	ca. 200,000 pg/ml* [122]	ca. 6,500 pg/ml* [11]	532.6 ± 71.77 ng/ml^c^ [101]	n/a
Control serum ante-mortem	Control serum post-mortem	1.70 ± 0.41 µkat/l [108]	37.8–89 µkat/l [23]	40–300 ng/ml [124]	1,750–2,220 ng/ml [23]	277,860 ± 28,110 pg/ml [122]	ca. 3,000–7,500 pg/ml* [11]	178.0 ± 19.83 ng/ml^c^ [101]	n/a

^*^Value read from graph; *a*, pediatric study cohort (age range 2–17 years); *b*, plasma value; *c*, sampling time not specified; *d*, TBI survival time not stated; *n/a*, not available; *r*, correlation index

**Table 3:** A summary of the selected forensically used traumatic brain injury (TBI) biomarker (MAPT) is given. R values are only provided if the *p*-value of that correlation was significant (≤ 0.05) and the values were stated in the related studies

		MAPT	
Molecular weight [kDa]		48–68 [125]	
Expression		Associated with microtubules in neurons, astrocytes and oligodendrocytes [126, 127], peripheral nerves [128]	
Functions		Cell signaling, synaptic plasticity, regulation of genomic stability [127]	
Reason for biomarker level change within compartment after traumatic head impact		Hypothesis for blood: Diffusion across disrupted BBB from CSF [129]	
Age-dependency		n/a	
Sex-dependency			
H-index-dependency			
Intensive care procedure/rescue procedure-dependency			
In-vitro freeze-thaw-cycle-dependency		CSF: stable for at least 6 cycles at − 80 °C [130]	
In vitro biomarker stability		CSF: stable for at least 22 days when stored at − 80 °C, 4 °C, or 18 °C, level decrease after 12 days when stored at 37 °C [130]	
Post-mortem interval correlation		Not investigated before	
TBI CSF ante-mortem	TBI CSF post-mortem	0.08–0.14 ng/ml [131]	48.43 ± 8.33 ng/ml^d^ [132]
Control CSF ante-mortem	Control CSF post-mortem	0.19 ± 0.06 ng/ml [133]	3.84 ± 0.31 ng/ml^d^ [132]
TBI serum ante-mortem	TBI serum post-mortem	0.24 ± 0.39 ng/ml^d^ [132]	22.42 ± 16.59 ng/ml^d^ [132]
Control serum ante-mortem	Control serum post-mortem	0.01 ± 0.02 ng/ml (undetectable in 9/10 cases) [132]	1.10 ± 0.31 ng/ml [132]

^*^Value read from graph; *a*, pediatric study cohort (age range 2–17 years); *b*, plasma value; *c*, sampling time not specified; *d*, TBI survival time not stated; *n/a*, not available; *r*, correlation index

### S100 calcium-binding protein B

#### Cause of death determination

CSF concentrations of S100B were significantly higher in TBI fatalities compared to controls (isolated torso trauma, cerebrovascular injury, and sudden natural deaths) [30, 31]. Recently, it was stated that a fatal acute TBI (survival time less than 2 h) can be detected with 79% accuracy and 97% specificity in post-mortem CSF when the S100B concentration reaches a threshold of 2267 ng/ml [26]. In serum, S100B was significantly higher in TBI cases compared with isolated torso traumas but was not statistically different from cases with cerebrovascular insufficiency and sudden natural deaths in a small sample size investigating 17 TBI fatalities and 23 controls [30]. However, all aforementioned controls were not statistically different from TBI fatalities in serum in a larger sample size of 45 TBI fatalities and 47 controls [31]. Serum S100B levels were significantly correlated with the severity of head injury [7]. It was observed that post-mortem serum S100B levels were also significantly elevated in fatalities with non-TBI-related brain injuries such as strangulation or hanging [7]. Also, significantly higher S100B concentrations were observed in serum of TBI fatalities compared to isolated torso traumas but not cerebrovascular injuries and sudden natural deaths [30].

#### Survival time estimation

CSF S100B levels were consistently increased compared to controls throughout survival times of up to 10 days [30, 31]. CSF S100B levels > 10,000 ng/ml were observed only in TBI fatalities with survival times of at least 20 min and in every TBI fatality with survival times between 2 h and 5 days [30]. Serum levels of subacute TBI fatalities (survival times between 3 and 48 h) were significantly higher compared to acute (survival time of few seconds to 42 min) TBI fatalities [30]. Serum S100B levels decreased again 72 h after traumatic head impact [30].

### Neuron-specific enolase

#### Cause of death determination

CSF samples of NSE have been determined as reliable measurements of TBI fatalities as levels were significantly elevated in TBI cases when compared to controls that died from isolated torso traumas and sudden natural deaths but not from acute myocardial infarctions [31]. NSE values in *CSF* > 6000 ng/ml were only observed in TBI fatalities, but not in control fatalities (isolated torso trauma, cerebrovascular insufficiency, and sudden natural death) [30]. Both NSE CSF and serum levels were not statistically different in TBI fatalities that showed a macroscopically visible brain contusion (and intracerebral bleeding) compared to cases that only revealed intracerebral bleeding [31]. Serum levels of TBI fatalities were statistically non-different from control cases (isolated torso trauma, cerebrovascular insufficiency, and sudden natural death) [30].

#### Survival time estimation

CSF NSE values > 6000 ng/ml were exclusively detected in TBI cases with a survival time between 15 min and 5 days [30]. Recently, it was stated that a lethal acute TBI (survival time less than 2 h) can be detected with an accuracy of 83% and a specificity of 97% in post-mortem CSF when the NSE concentration reaches a threshold value of 599 ng/ml [26]. Peak CSF concentrations of NSE were reached within survival times of 3 to 4 days [31]. No significantly different CSF NSE concentrations between TBI fatalities and controls that died from hypoxia, sudden cardiac events, or miscellaneous causes were observed within a mean TBI survival time of 1 h [29]. For TBI fatalities with a maximum survival time of up to 2 h, CSF NSE levels were shown to be significantly higher compared to cases of diffuse cerebral hypoxia and isolated torso trauma, but not acute myocardial infarctions [31]. Serum levels of NSE did not correlate with the survival times of TBI fatalities on a statistically significant level [30].

### Glial fibrillary acidic protein

#### Cause of death determination

CSF and serum GFAP levels have been shown to be significantly increased in TBI fatalities compared to myocardial infarction and isolated torso trauma deaths, but not diffuse cerebral hypoxia [11, 13]. Post-mortem CSF GFAP levels are not specific for TBI fatalities, as they revealed a higher median for diffuse cerebral hypoxia compared to acute (survival times of less than 2 h) and delayed (survival times between 72 and 456 h) TBI fatalities [11]. When the GFAP CSF level exceeds 385.5 ng/ml, a fatal TBI can be diagnosed with a sensitivity of 71.1% and a specificity of 71.4% [11]. For fatal TBIs with a survival time of fewer than 2 h, a GFAP CSF concentration of 134 ng/ml discriminates a TBI fatality from control fatalities (acute myocardial infarction, diffuse cerebral hypoxia, and isolated torso trauma) with an accuracy of 78% and a specificity of 94% [26]. Contrary to the study of Ondruschka et al. [11], another post-mortem study revealed no differences in serum GFAP level between fatalities with macroscopically visible brain damage (including TBI) compared to control fatalities (cardiac cause, respiratory cause, intoxications, exsanguinations, or multi-organ failures) [27]. In serum, a fatal TBI can be diagnosed with a sensitivity of 76.2% and a specificity of 73.8% once the GFAP concentration surpasses 0.91 ng/ml [11]. Huge inter-individual variations were observed for both GFAP CSF and serum levels [11].

#### Survival time estimation

In CSF, the GFAP level peaks in the subacute group (survival time between 2 and 60 h). However, no significant difference was detected between the different TBI survival times neither in CSF nor in serum [11]. In serum, GFAP levels peak in acute TBI fatalities (survival times of less than 2 h) and, with increasing TBI survival times up to 456 h, approximate the concentrations of the control group (acute myocardial infarction, diffuse cerebral hypoxia, and isolated torso trauma) [11].

### Interleukin-6

#### Cause of death determination

IL-6 levels in CSF and serum are significantly higher in TBI fatalities compared to non-infectious controls for which the survival time was assumed to be zero such as atraumatic hypoxic brain damage or acute myocardial infarction [11, 21]. When IL-6 levels of TBI fatalities were compared to fatalities that died from isolated torso trauma, CSF but not serum levels were significantly higher [11]. Recently, it was shown that a lethal acute TBI (survival time less than 2 h) can be detected with an accuracy of 86% and a specificity of 96% in post-mortem CSF when the IL-6 concentration reaches a threshold value of 99.1 pg/ml [26]. Trauma fatalities including TBI fatalities revealed significantly higher serum values compared to atraumatic deaths resulting from atraumatic causes of death as well as natural deaths [22].

#### Survival time estimation

With regards to the TBI survival time, no statistically significant differences of IL-6 levels were detected in post-mortem CSF and serum samples within an investigated survival time span of at least 3 days [11]. However, CSF IL-6 levels of more than 100,000 pg/ml were only detected in TBI fatalities with a survival time of more than three days [23].

### Lactate dehydrogenase

#### Cause of death determination

LDH CSF levels of TBI fatalities (all survival times pooled between a few seconds and 19 days) are significantly higher compared to controls that died from isolated torso trauma, diffuse cerebral hypoxia, or acute myocardial infarction [23]. Also, LDH CSF levels were higher in TBI fatalities compared to fatalities due to hypoxia, sudden cardiac death, or natural and non-natural deaths that could not be attributed to any of the former [28, 29]. Recently, it was stated that a lethal acute TBI (survival time less than 2 h) can be detected with an accuracy of 81% and a specificity of 97% in post-mortem CSF when the LDH concentration reaches a threshold value of 16.71 ukat/l [26]. Serum LDH levels of TBI fatalities (all survival times between a few seconds and 19 days pooled) were only higher compared to isolated torso traumas, but not for diffuse cerebral hypoxia or acute myocardial infarctions [23]. In vitreous humor, LDH levels were higher in TBI fatalities compared to sudden cardiac deaths, but lower than hypoxia-related deaths or natural and non-natural deaths that could not be classified as TBI-related, hypoxia, or sudden cardiac deaths [28]. However, it was not mentioned whether the former results in vitreous humor were statistically significant [28].

### Survival time estimation

For CSF, LDH levels were shown to be stable for survival times between a few seconds and 19 days in one study [23] but decreased in another study that investigated a TBI fatality group with a mean survival time of 1 h [29]. Serum LDH levels have not been stated to vary on a statistically significant level for TBI fatalities with survival times ranging from a few seconds to 19 days [23]. No statistically significant survival time dependence was stated for vitreous humor levels of LDH [29].

### Ferritin

#### Cause of death determination

CSF ferritin levels of TBI fatalities were significantly higher compared to each of the following fatality groups: isolated torso trauma, diffuse cerebral hypoxia, and acute myocardial infarction [23]. CSF ferritin levels of > 8.0 mg/l were only reached by TBI fatalities but none of the aforementioned controls fatalities [23]. Recently, it was stated that a lethal acute TBI (survival time less than 2 h) can be detected with an accuracy of 87% and a specificity of 96% in post-mortem CSF when the ferritin concentration reaches a threshold value of 1.73 mg/l [26]. In serum, the pooled TBI fatalities were only significantly higher compared to diffuse cerebral hypoxia fatalities, but not for acute myocardial infarctions of isolated torso traumata [23].

#### Survival time estimation

Both CSF and serum levels of ferritin were significantly higher for TBI fatalities with a survival time of more than 72 h (maximum 19 days) compared to survival times between a few seconds and 43 h [23]. A CSF ferritin level of > 30.0 mg/l was only reached after a minimum TBI survival time of 2 h [23].

### Brain-derived neurotrophic factor

#### Cause of death determination

CSF BDNF values are discriminative between TBI fatalities and fatalities that died from diffuse cerebral hypoxia and acute myocardial infarction but not from isolated torso trauma [11]. A TBI fatality can be diagnosed post-mortem with a sensitivity of 71.0% and a specificity of 83.3% when a CSF BDNF level of 29.0 pg/ml is reached [11]. Recently, it was noted that a lethal acute TBI (survival time less than 2 h) can be detected with an accuracy of 86% and a specificity of 96% in post-mortem CSF when the BDNF concentration reaches a threshold value of 11.1 pg/ml [26]. Serum BDNF values of TBI fatalities were statistically non-different from the aforementioned control groups [11]. Huge inter-individual variations were observed for BDNF CSF and serum levels [11].

#### Survival time estimation

Both CSF and serum levels of TBI fatalities revealed the highest median levels in acute TBI fatalities with a survival time between a few seconds and 107 min [11]. The CSF and serum values of BDNF decreased with increasing survival times [11]. However, neither CSF nor serum levels revealed statistically significant BDNF level changes between the trauma survival time groups (survival times between a few seconds and 456 h), which renders the marker not useful for survival time estimations [11].

### Neutrophil gelatinase-associated lipocalin(lipocalin-2)

#### Cause of death determination

NGAL CSF levels of TBI fatalities were significantly higher compared to each of the following control fatalities: isolated torso trauma, diffuse cerebral hypoxia, and acute myocardial infarction [11]. A CSF NGAL value of 1050.5 ng/ml detects a lethal TBI with a sensitivity of 72.7% and a specificity of 89.7% [11]. Huge inter-individual variations were observed for NGAL CSF levels [11]. A lethal acute TBI (survival time less than 2 h) can be detected with an accuracy of 84% and a specificity of 94% in post-mortem CSF when the NGAL concentration reaches a threshold value of 334.4 ng/ml [26]. Post-mortem NGAL serum measurements are not described yet.

#### Survival time estimation

CSF values of NGAL revealed the highest median values for survival times between 2 and 72 h; however, no statistically significant differences were observed regardless of the investigated survival times between a few seconds and 456 h of TBI fatalities [11].

### Microtubule-associated protein Tau

#### Cause of death determination

Post-mortem CSF, serum, urine, and saliva levels of MAPT were significantly higher in a group consisting of TBI fatalities and fatalities with a suspected TBI as a co-morbidity based on macroscopic signs compared to a control group that consisted of deaths from sudden cardiopulmonary failures [33]. However, no statistically significant MAPT levels were found in vitreous humor between the aforementioned groups [33].

## Discussion

Accurate and reliable evidence collection is an essential component of forensic medicine and thus makes an important contribution to the proper function of the legal system. Forensic biochemistry is an accepted part of particular forensic investigations [8] and research efforts have increased dramatically in the last decade [11–13, 23, 24, 26, 30, 33]. This review paper summarized the current literature on post-mortem biomarkers in TBI-related forensic questions. A critical consideration of the summarized findings and an outlook on this forensic niche is provided below.

### The value of forensic biomarkers to determine a TBI as the cause of death

The summary given here revealed that several biomarkers discriminate between TBI fatalities and several different control fatalities at a statistically significant level. This indicates that forensic biochemistry is a promising field to provide additional objective data to determine TBI as the cause of death. Regarding this, several points have to be critically discussed based on this given review. Ideally, if a biomarker reaches a particular threshold value in a certain compartment, a lethal TBI can be diagnosed with 100% sensitivity and specificity, respectively. However, none of the biomarkers used in forensic science is specific for fatal TBI but shows significant changes in marker values in other fatalities as well, which is commonly much higher when compared to living subjects. Moreover, this summary highlights that each individual biomarker was statistically dependent on at least one co-factor such as PMI, hemolysis, or whether neurosurgery was performed. Together with unpredictable peri- and post-mortem changes including untraceable biomarker concentration changes in the respective compartments [134], the TBI-related biomarkers show enormous standard variations in both TBI fatalities and controls, which usually overlap. Therefore, none of the biomarkers used forensically to date is able to distinguish between TBI and non-TBI fatalities based on the concentration of a particular biomarker in 100% of cases. However, several cut-off values, mainly in CSF, have already been reported to corroborate the suspicion of a lethal TBI together with other post-mortem investigation results rather than to prove it independently [11, 23, 26, 30]. Indeed, the selection and careful categorization of control fatalities within the studies on post-mortem TBI-biomarkers are of special interest. For example, serum IL-6 can discriminate TBI fatalities from fatalities due to acute myocardial infarctions and diffuse cerebral hypoxias, but not from isolated torso traumas [23]. This information can provide valuable objective evidence when serum IL-6 concentration is considered together with other autopsy findings, e.g., when an isolated torso trauma can be excluded. Therefore, it is of higher importance to compare TBI fatalities with homogenous individual control groups rather than with a pooled control group of all non-TBI cases. When thresholds are set by individual studies, this is essentially against the selected (or available) control deaths. However, this literature review revealed that there is considerable variation in the definition of control deaths between studies, potentially affecting the respective results and conclusions. Apart from the voluntary allocation of fatalities to the TBI and control groups, a TBI fatality, or at least a fatality with a TBI as a confounding cause, could easily end up in the control group in some studies, affecting the results, which is more likely in cases without macroscopic correlates of the traumatic event against the head. Lastly, using forensic biochemistry might be challenging to reliably discriminate between TBI fatalities and control cases whenever the entire cohort is considered. However, it seems to be realistic to define “extreme” biomarker levels that are just achieved by TBI fatalities, which essentially means defining upper cut-off values that reflect 100% specificity with poor sensitivity. The CSF NSE values > 6000 ng/ml, which have just been reached in TBI fatalities [30], are an example of such an “extreme” cut-off value. It has to be mentioned that threshold values apply only when the same laboratory testing setup, and a measurement kit is used as in the respective study. However, this has to be validated against a broad variety of control fatalities to be valid with reasonable certainty. Some of the aforementioned studies revealed difficulties in defining cut-off values due to different measurement methods and inter-individual differences.

### Post-mortem biomarkers for TBI survival time estimations - are they useful?

The previous research on TBI-related post-mortem biomarkers uncovered the potential of several biomarkers in various compartments to discriminate between different survival times of TBI fatalities. The factors that influence the cause of death determination can equally be listed for survival time estimations, explaining why particular biomarker levels that pinpoint certain survival times are lacking to date or, in fact, most likely impossible to achieve. However, the here given literature summary observed a trend that certain post-mortem TBI biomarkers can indicate minimum survival times if high biomarker values are reached [23, 30]. CSF levels of > 10,000 ng/ml for S100B [30], > 6000 ng/ml for NSE [30], > 30 mg/l for ferritin [23], and > 100,000 pg/ml for IL-6 [23] were only observed for minimum survival times of 20, 15, 120 min, and 3 days, respectively. Since a tendency for low biomarker concentrations can be suspected for short survival times [23], more such cut-off values for determining a minimum survival time probably exist for other compartments besides CSF but have not received sufficient attention so far. Future research on TBI survival time estimations using post-mortem biomarkers should report cut-off values for minimum survival times to further explore the potential of whether post-mortem biochemistry can provide reliable cut-off values for minimum survival times in TBI fatalities.

### The role of post-mortem biomarkers in time since death estimations of TBI fatalities

Several biomarkers of various compartments correlated with the PMI [23, 28, 30, 31], being the fundamental requirement to be used for time since death estimations. However, this correlation was largely attributed to the increasing hemolysis occurring with increasing PMI. Future studies on fluid TBI-related biomarkers should provide additional quantitative information on the correlation between the biomarker concentrations and the PMI. Moreover, the use of cut-off values should be explored for PMI correlations as well. In light of the potentially inevitable influence of the progressive hemolysis in post-mortem samples, cut-off values for particularly short PMIs yielding low biomarker concentrations seem most promising in this regard. However, given the currently available information, post-mortem biomarkers seem to be of no merit for time since death estimations of TBI fatalities.

### The present and future of post-mortem TBI-related biomarkers

This literature review demonstrated the potential of post-mortem biomarkers to provide objective evidence for cause of death determinations and survival time estimations of TBI fatalities. However, forensic biochemistry, as a promising investigative branch of forensic medicine, is still at the very beginning and data on particular causes of death such as TBI fatalities are scarce. Therefore, it is yet too early to include current observations into the daily routine without further verifications that respect detected pitfalls such as the influence of perimortem procedures or hemolysis on the biomarker levels. Equally, TBI-related biomarkers that did not reveal sufficient potential to provide additional information for forensically relevant investigations should not be neglected too soon as these results could have been biased by limited sample sizes or the inaccurate allocation of TBI fatalities to the control group and vice versa. LDH and MPAT were the only two biomarkers that were investigated in post-mortem samples other than CSF, serum, or the brain so far [28, 33]. Especially, MPAT demonstrated the potential of discriminating TBI fatalities from controls in urine and saliva and these two compartments should be further investigated using other biomarkers. Moreover, other promising clinically relevant TBI biomarkers should be investigated in post-mortem body fluids in the future. These include for example αII-spectrin breakdown products, myelin basic protein, neurofilament proteins, ubiquitin C-terminal hydrolase-L1, tumor necrosis factor alpha, or interleukin-1B [19]. Ideally, all observations on post-mortem biomarkers including but not limited to the ones of TBI fatalities should be collected in forensic biochemical databases to further explore the opportunities and challenges of this emerging post-mortem field allowing for collective sample sizes that surpass the ones of individual departments by far.

Recent pioneering works regarding the consideration of mi-RNAs [135] or the entity of metabolites (metabolome) [136, 137] to prove lethal TBIs in a forensic setting should be further explored. Using a combination of six different mi-RNAs, it was possible to discriminate TBI cases from controls that were free of neurological symptoms [135]. Even though the former study was based on ante-mortem blood samples, the six candidate mi-RNAs were identified and validated on 38 post-mortem brain tissues before [135, 138]. Groups of metabolites were shown to be relevantly elevated in TBI fatalities compared to controls in post-mortem CSF [136]. Future studies will tell whether mi-RNAs and metabolites are superior to the forensically used TBI biomarkers that were discussed in this review and if/how much a combination of all these fluid biomarker groups can benefit forensic practice.

### Limitations

Based on the selected search criteria, especially the selected search terms, some relevant articles might have been missed. Initially, the articles were retrieved through title screening, which might have led to an exclusion of relevant articles based on inappropriate title selection by the respective authors or misinterpretation by the authors of this given literature review. Location bias might have led to an oversight of articles in less accessible journals.

## Conclusions

Forensic TBI-biomarkers are an emerging and promising resource to provide objective evidence for cause of death determinations and survival time estimations. However, all TBI-biomarkers that were forensically investigated to date are unspecific for TBIs and only allow for particular information such as detections of TBI fatalities with poor sensitivity or minimum survival time estimations. Future research on forensic biomarkers requires a strict separations of TBI fatalities and control groups with sufficient sample sizes, the exploration of the current biomarkers in additional compartments such as urine, saliva, and vitreous humor, the addition of further clinically promising biomarkers to the forensic field, and the in-depth forensic exploration of promising biomarker categories such as metabolites or mi-RNAs.
